# Whole-Body Cryotherapy: Potential to Enhance Athlete Preparation for Competition?

**DOI:** 10.3389/fphys.2019.01007

**Published:** 2019-08-06

**Authors:** Emily M. Partridge, Julie Cooke, Andrew McKune, David B. Pyne

**Affiliations:** ^1^Research Institute for Sport and Exercise Science, University of Canberra, Bruce, ACT, Australia; ^2^Faculty of Health, University of Canberra, Bruce, ACT, Australia; ^3^Collaborative Research in Bioactives and Biomarkers (CRIBB) Group, University of Canberra, Bruce, ACT, Australia; ^4^Discipline of Biokinetics, Exercise and Leisure Sciences, School of Health Sciences, University of KwaZulu-Natal, Durban, South Africa

**Keywords:** potentiation, cooling, performance, hormone, cryotherapy

## Abstract

The final hours of preparation before competition are important for performance. Recovery, preparation and warm up protocols are evolving continuously and include passive and active modalities often developed by “trial and error” approaches. Passive modalities, such as whole-body cryotherapy (WBC), have the potential to enhance both recovery and preparation. Whole-body cryotherapy has generally been used within a recovery setting after competition or strenuous training for athletes, and in clinical settings for the general population. However, the acute hormonal, anti-inflammatory, perceptual and psychological responses yielded by a single, or repeated, bouts of WBC indicate that this practice could enhance an athlete’s competition readiness when used alongside traditional elements of active warm-ups in the hours before competition in addition to aiding recovery in the hours after. Here we summarize and evaluate the acute effects of WBC exposures on physiological, performance and perceptual responses, and examine the likelihood these responses could theoretically translate into enhanced athletic performance. The potential to enhance an athlete’s performance using acute passive WBC exposure is a novel intervention that requires further investigation.

## Introduction

In high-performance sport, coaches and sports scientists strive to identify novel interventions to enhance performance. Some of these interventions are employed to optimize the physical, psychological and behavioral state of an athlete prior to competition or training (Sands et al., 2017). The window of preparation prior to competition is vital to subsequent athletic performance with the goals of enhancing oxygen uptake, cardiac output, blood flow to skeletal muscle, neuromuscular activation and mental alertness (McGowan et al., 2015).

The warm-up typically involves both passive and active elements which can enhance exercise performance (McGowan et al., 2017). Active warm ups are used to increase body temperature leading to a higher muscle metabolism, enhanced oxygen uptake and subsequent cardiac output (Kilduff et al., 2013). Passive warm up requires less energy expenditure and center on various psychological and motivational techniques, or cooling/heating interventions to prepare the athlete (Cook and Crewther, 2012; Cook et al., 2013). With technological advances, preparation through passive modalities is becoming more common for player management and performance enhancement (McGowan et al., 2016). While every team, coach and performance manager have individualized methods and preferences for their athletes or players, newer and more advanced methods of performance enhancement are evolving.

Whole-body cryotherapy (WBC) is a relatively new clinical intervention used to alleviate pain in inflammatory conditions and more recently as a recovery modality in elite athletes (Banfi et al., 2010). Given the extremely cold air temperatures of –120° to –150°C reached in short 2–3 min exposures, it can elicit strong physiological responses in users (Zalewski et al., 2013). Responses linked with exercise and sporting performance include modulations in testosterone and cortisol levels; autonomic nervous system activation; and subsequent post-activation potentiation (PAP) effects (Christophe et al., 2013; Schaal et al., 2015; Lombardi et al., 2017; Russell et al., 2017). The likely effects of WBC use prior to exercise, and the underlining mechanisms, need clarification to inform the use by athletes before competition.

The purpose of this perspective is to explore the acute effects of WBC exposure on physiological factors, post-activation potentiation and exercise performance. This information should clarify whether pre-game WBC could enable athletes to be in a greater state of readiness for competition.

## Whole-Body Cryotherapy for Clinical and Recovery Applications

Interest in WBC has increased rapidly over the past decade with two main applications identified: the physical and mental benefits of exposure in clinical patients and to promote recovery after exercise (Bouzigon et al., 2016). WBC has been used in patients with fibromyalgia over a 3-week period of exposure every second day yielding a decrease in pain on a visual analog scale (VAS) compared with a control group (Rivera et al., 2018). Patients with ankylosing spondylitis participated in an 8-day intervention of WBC exposure in which proinflammatory cytokines and overall disease activity decreased in the WBC group compared to an exercise control group (Straburzynska-Lupa et al., 2018). WBC in a clinical setting is generally used over a period of at least 7 days rather than acute bouts to potentially elicit greater effects on the underlying condition. In the sporting context, promotion of recovery is the main use for athletes at present. These two broad applications likely share similar underlying physiological, psychological and perceptual mechanisms.

The most common use of WBC identified in sport is currently as a recovery modality after high intensity exercise and/or competition for athletes. Marketed as a new means for muscle and physical recovery, this interest has accelerated in recent years in high-performance sport. Researchers have identified how WBC can improve recovery post-exercise by minimizing sensations of delayed onset muscle soreness (DOMS) or exercise-induced muscle damage (EIMD). These outcomes are largely due to anti-inflammatory effects identified in initial research in alleviating pain in inflammatory conditions (Lombardi et al., 2017). More specifically, WBC increased the concentration of the anti-inflammatory cytokine interleukin (IL)-10, and decreased both the pro-inflammatory cytokine IL-2 and chemokine IL-8 (Banfi et al., 2010). Limiting pain-related symptoms elicited by DOMS or EIMD could lead to a greater force output from skeletal muscle in athletes using WBC.

Whole-body cryotherapy has also been shown to yield changes in the concentrations of muscle enzymes including serum creatine kinase (CK). CK is typically used to monitor muscle damage, recovery and possible overtraining in athletes (Banfi et al., 2010). The acute use of WBC exposure on alternate days over a 1 week period by professional rugby players induced a 40% decrease in the circulating concentration of CK during a regular training workload week (Banfi et al., 2009). While changes in CK can be caused by various physiological mechanisms, there appears to be a link between WBC exposure and a decrease in CK concentration. However, the question remains whether pre-event WBC can be used in a novel application to enhance player performance and physical wellbeing rather than solely to improve recovery in individuals.

## Whole-Body Cryotherapy as a Means of Invoking Post-Activation Potentiation in Athletes

Post-activation potentiation (PAP) is a physiological phenomenon describing acute changes in muscular power output in the 1–3 h post-priming activity and may be beneficial for athletes looking to improve performance (Hodgson et al., 2005). In mechanistic terms, a PAP effect occurs through phosphorylation of myosin regulatory light chains, which makes actin and myosin more sensitive to Ca^2+^ (Hodgson et al., 2005). PAP is more commonly understood to occur using complex movements incorporating concentric weight training with high loads followed by plyometric exercises involving the same biomechanical movement demands (Docherty and Hodgson, 2007). Opposing this however, improvements in countermovement jump muscular performance have been shown to elicit no changes in PAP or neuromuscular function when performed post-heavy resistance training in male athletes (Thomas et al., 2017).

The use of WBC prior to exercise may have a beneficial effect on power output related to the alleviation of pain and sense of fatigue. One of the most apparent effects of declining muscle output in force-generating capabilities is the sensation of pain and reduced muscle fiber recruitment. However, the idea of inducing PAP from the exposure to WBC in athletes introduces other physiological mechanisms that could enhance performance capabilities. The influence that hormones such as testosterone, cortisol and blood catecholamines have on an individual’s athletic capacity need to be investigated more thoroughly.

It appears that WBC exposure could elicit a PAP response when utilized prior to exercise or competition. Over a period of 5 days, twice daily exposure to WBC at –120°C elicited 7% better stroke effectiveness in 15 elite tennis players, measured through shot accuracy and speed (Ziemann et al., 2012). Another study reported that 3 min of WBC at –110°C failed to effect force output and muscle soreness when measured immediately and 15 min after exposure (Costello et al., 2012). It is difficult to compare studies directly with methodological differences involving the WBC exposure temperature, duration, before/after exercise and/or the type of exercise utilized. However, neither study outcomes should be discredited outright, and need developing further to distinguish applications in sport.

## Hormonal Changes Induced by Whole-Body Cryotherapy

### Testosterone

Considering the goal of enhancing athlete wellbeing prior to competition, hormones with an established circadian rhythm could be a key mechanism manipulated through WBC exposure. Testosterone is a primary anabolic hormone that plays an important role in generation of muscle mass and strength which are both vital for athletic performance. Salivary testosterone levels in elite female athletes have been associated with an individual’s natural desire to compete and motivation in training and readiness for competition (Crewther and Cook, 2018). WBC exposure has the potential to elicit acute exercise-like effects in hormone concentrations following exposure, which could partly explain the benefits of this intervention. A single bout of WBC in men undertaken within 20 min of repeated sprint exercise elevated free testosterone levels by 28% for 24 h peaking at 2 h post-cryotherapy compared with a control group (Russell et al., 2017). Similarly, serum testosterone in elite male tennis players increased by 13% over a 2-week period of twice daily WBC exposure (Ziemann et al., 2012). As biomarker concentrations are relatively variable in both inter- and intra-subject settings, carefully defined and executed studies are needed to clarify the possible benefits of WBC.

Free testosterone plays an important role in motivation and social aspects of an athlete’s overall demeanor. Hormonal fluctuations can be promoted with visual feedback, in the context of motivational videos (categorized as erotic, aggressive or sports-specific in nature) before voluntary squats improved 3RM maximal squat weight by 2–6 kg (Cook and Crewther, 2012). Acute bouts of WBC could reduce the levels of perceived soreness, and fatigue, and increase aggression levels in high-performance athletes. Implementation of WBC to enhance the level of natural testosterone in an individual could be beneficial to motivation levels and subsequent performance. This is especially important in male athletes given the large circadian rhythm of testosterone concentration and that many sports are played in the afternoon to late evening. Offsetting the reduction in, or augmenting, testosterone levels would be of benefit to athletes.

### Cortisol

Stress on the body elicits multiple physiological responses including activation of the hypothalamic-pituitary-adrenal (HPA) axis and secretion of cortisol. The relationship between WBC, exercise and stress-related cortisol levels have been investigated previously although results are somewhat inconsistent and inconclusive. Long-term repeated bouts of WBC over a 12 week period yielded a 23–32% decrease in cortisol compared to initial or baseline levels in healthy females (Leppäluoto et al., 2008). In a shorter timeframe, over a 5-day period of repeated WBC exposures preceding exercise in elite rowers the concentration of cortisol increased 13–25% more in the control group than the WBC intervention (Wozniak et al., 2013). Given the acknowledged influence of high cortisol levels on decreased performance, WBC exposure diminishing this effect could have positive performance consequences.

While a marked decrease in cortisol concentration is regarded as a positive influence on performance outcomes, the ratio of testosterone to cortisol (T:C) is also of interest for exercise performance. An inverse T:C relationship has been suggested as a useful means of identifying the status of hormonal balance in athletes (Strahorn et al., 2017). The T:C ratio was 7% higher in pre-game measures where the outcome was a win for a rugby team than before a team loss (Gaviglio et al., 2014). Over a 7-day period of WBC exposures twice per day, 25 elite rugby players exhibited a 43% decrease in salivary cortisol concentration, but associated with a 47% increase in salivary testosterone (Grasso et al., 2014). Despite these outcomes further work is required to identify which interventions can enhance the T:C ratio under a variety of situations across different sports. Furthermore, with little evidence to support the hormonal response to single or repeated bouts of WBC on the T:C ratio, it is important to clarify the pattern and time course of these changes.

### Alpha-Amylase and Catecholamines

Salivary alpha-amylase (sAA) has been proposed as a biomarker for stress-related changes in the body as a surrogate measure of blood catecholamine concentration (Nater and Rohleder, 2009), particularly dopamine, norepinephrine, and epinephrine (Lim, 2016). Acute heavy resistance exercise increased blood catecholamines by an average of 270% comparing pre-post resistance training values in 10 trained men (French et al., 2007). In response to a single WBC exposure of 3 min at –110°C in a healthy male population, plasma norepinephrine levels increased by 76% compared with a control group (Christophe et al., 2013). Abrupt and intense immersion into a cold environments can elicit efferent sympathetic nervous system activity to skeletal muscle, producing an increase in heart rate and presumably noradrenaline release (Kregel et al., 1992). This mechanism could be one explanation for how WBC could impact favorably on an athletes’ preparation and subsequent readiness.

It has been demonstrated that sAA levels can increase from both physically demanding and psychologically stressful situations (Chatterton et al., 1996). Activation of sympathetic neural pathways at the onset of dynamic exercise has a major effect on cardiovascular, hormonal and metabolic responses, and the subsequent muscular performance of individuals (Christensen and Galbo, 1983). However, increases in sAA are evident following repeated WBC exposures with a study illustrating a plateau in sAA levels following a 2 week period of daily WBC exposure for 3 min at –110°C compared to a 26% decrease in the control group (Schaal et al., 2015). If further research can identify a relationship between WBC exposure and sAA (and blood catecholamine release), this could explain any marked reduction in muscular fatigue and enhancement of muscle priming effects.

### Psychological Wellbeing

The effects of WBC exposure on the psychological wellbeing of participants has not been investigated thoroughly. In one instance, WBC exposure of 3 min at –110°C over 10 sessions lifted overall mood by ∼13% and wellbeing using the World Health Organization Quality of Life-Bref questionnaire and the Psychological General Well-Being Index (Szczepanska-Gieracha et al., 2014). Although different to WBC exposure, cold water immersion post-exercise reduced perceived lower leg muscle soreness by 34% which can play a positive role in athletic performance and well-being (Ahokas et al., 2019). In a sporting context, the wellbeing and mentality of high-performing athletes is universally acknowledged as an important factor regulating performance (Strahorn et al., 2017). Interventions are often implemented to ensure an individual is in the best possible state of physical, psychological and behavioral readiness for competition (Serpell et al., 2018). The typical athletic profile is multi-dimensional, with championship performance occurring as a function of psychological prowess, interpersonal support and effective training strategies (Burns et al., 2018). Psychological wellbeing needs to be regarded as an independent factor pertaining to performance rather than a by-product of an athlete’s physical readiness.

An athlete’s level of self-perceived stress and physical discomfort from prior training has been linked to reduced performance in multiple settings (Bagheri et al., 2018). Monitoring an athlete’s overall wellbeing and self-awareness raises the question whether reducing pain sensation and fatigue awareness has the potential to be performance enhancing. High-level basketball players using cold-water immersion directly after competition reduced 34% of perceived lower leg muscle fatigue after 24 h, associated with a 45% increase in jump performance (Delextrat et al., 2013). The influence of other recovery or maintenance strategies on an athlete’s psychological wellbeing has been documented, although no studies have directly evaluated the use of WBC on physiological factors in athletes.

## Applications and Recommendations

Anecdotal reports from strength and conditioning coaches and athletes indicate that WBC could have a performance enhancement capability when used ∼3 h prior to competition. This timeline differentiates the traditional use of WBC as a recovery tool from the novel implementation of a performance enhancing technique in the preparation period. While this proposition is attractive, the high-level athlete is continually in a variable pattern of physical fatigue and recovery between training and competitive performance. This is particularly the case in team sports playing one or more games per week in a seasonal competition. Recovery can be provided in various forms including massages, foam rolling, trigger pointing or other pain relief techniques. These modalities are commonly used in the hours preceding competition to alleviate pain and enable the athlete to in the best possible state of readiness to perform pain free from muscle aches. In this context, the use of WBC prior to competition alongside active warm-up modalities, has promise to complement these traditional methods of preparing for competition.

Athletes involved in sport having multiple competitions on the same day or over a longer period of 7 + days may benefit from using WBC before their competitions, event or game. Recent anecdotal reports indicate that athletes are using WBC at half-time during competition or in-between same day heats or rounds of a tournament. With the availability of mobile cryotherapy chambers slowly becoming more accessible, it could be become practically feasible for athletes to use the WBC treatment both pre-competition, and between halves or games played on the same day, to promote performance. During competitions with heat protocols in place for player safety during hot weather, this treatment could potentially be more beneficial than more traditional cold-water immersion techniques.

As WBC is a relatively new treatment in the private and commercial market, the viability and effectiveness of this practice in a sporting setting are yet to be fully described. The lack of availability and cost of WBC in the public domain is a major limitation for athletes gaining access to the treatment. While large professional teams can afford the expenses associated with owning a WBC chamber or facility themselves, individual and unfunded athletes may be at a disadvantage. Studies are needed that compare the effectiveness of different combinations of warm-up interventions from performance, physiological, accessibility and cost-benefit perspectives.

## Conclusion

WBC shows the potential as a novel warm-up intervention prior to competition by high-performance athletes when integrated with the traditional elements of active warm-ups. It appears that the physiological responses associated with acute and/or chronic exposure to WBC can elicit effects that could promote athletic performance when employed before competitive events. The potentiating effects of hormonal changes, reductions in body temperatures and perceived soreness and fatigue, all point to the potential of WBC as a warm-up activity. However, more evidence is required to prepare recommendations for the high-performance sporting community.

## Data Availability

This manuscript contains previously unpublished data. The name of the repository and accession number(s) are not available.

## Author Contributions

EP: conception and design, manuscript construction and critical revision, drafting the manuscript, final approval, and agreement on all aspects of the work. JC, AM, and DP: conception and design, drafting the manuscript, final approval, and agreement on all aspects of the work.

## Conflict of Interest Statement

The authors declare that the research was conducted in the absence of any commercial or financial relationships that could be construed as a potential conflict of interest.

## References

[B1] Ahokas K. E. Ihalainen K. J. Kyröläinen A. H. Mero A. A. (2019). Effects of water immersion methods on postexercise recovery of physical and mental performance. *J. Strength Cond. Res.* 33 1488–1495. 10.1519/JSC.0000000000003134 31008862

[B2] Bagheri R. Pourahmadi M. R. Hedayati R. Safavi-Farokhi Z. Aminian-Far A. Tavakoli S. (2018). Relationships between hoffman reflex parameters, trait stress, and athletic performance. *Percept. Mot. Skills* 125 749–768. 10.1177/0031512518782562 29909738

[B3] Banfi G. Lombardi G. Colombini A. Melegati G. (2010). Whole-body cryotherapy in athletes. *Int. J. Sports Med.* 40 509–517. 10.2165/11531940-000000000-00000 20524715

[B4] Banfi G. Melegati G. Barassi A. Dogliotti G. Melzi D’eril G. Dugué B. (2009). Effects of whole-body cryotherapy on serum mediators of inflammation and serum muscle enzymes in athletes. *J. Therm. Biol.* 34 55–59. 10.1016/j.jtherbio.2008.10.003

[B5] Bouzigon R. Grappe F. Ravier G. Dugue B. (2016). Whole and partial-body cryostimulation/cryotherapy: current technologies and practical applications. *J. Therm. Biol.* 61 67–81. 10.1016/j.jtherbio.2016.08.009 27712663

[B6] Burns L. Weissensteiner J. R. Cohen M. (2018). Lifestyles and mindsets of olympic, paralympic and world champions: is an integrated approach the key to elite performance? *Br. J. Sports Med*. 53:bjsorts-2018-099217. 10.1136/bjsports-2018-099217 30352862

[B7] Chatterton R. T. Vogelsong K. M. Lu Y. Ellman A. B. Hudgens G. A. (1996). Salivary alpha amylase as a measure of endogenous adrenergic activity. *J. Clin. Physiol.* 16 433–448. 10.1111/j.1475-097x.1996.tb00731.x 8842578

[B8] Christensen N. J. Galbo H. (1983). Sympathetic nervous activity during exercise. *Annu. Rev. Physiol.* 45 139–153. 10.1146/annurev.ph.45.030183.001035 6342511

[B9] Christophe H. Karine S. Yann Le M. François B. Jean-Robert F. Marielle V. (2013). Parasympathetic activity and blood catecholamine responses following a single partial-body cryostimulation and a whole-body cryostimulation. *PLoS One* 8:e72658. 10.1371/journal.pone.0072658 23991134 PMC3749989

[B10] Cook C. J. Crewther B. T. (2012). Changes in salivary testosterone concentrations and subsequent voluntary squat performance following the presentation of short video clips. *Horm. Behav.* 61 17–22. 10.1016/j.yhbeh.2011.09.006 21983238

[B11] Cook C. J. Crewther B. T. Kilduff L. P. (2013). Are free testosterone and cortisol concentrations associated with training motivation in elite male athletes? *Psychol. Sporst Exerc*. 14 882–885. 10.1016/j.psychsport.2013.08.001

[B12] Costello J. T. Algar L. A. Donnelly A. E. (2012). Effects of whole-body cryotherapy (−110°C) on proprioception and indices of muscle damage. *Scand. J. Med. Sci. Sports* 22 190–198. 10.1111/j.1600-0838.2011.01292.x 21477164

[B13] Crewther B. T. Cook C. J. (2018). A longitudinal analysis of salivary testosterone concentrations and competitiveness in elite and non-elite women athletes. *Physiol. Behav.* 188 157–161. 10.1016/j.physbeh.2018.02.012 29425972

[B14] Delextrat A. Calleja-Gonzalez J. Hippocrate A. Clarke N. D. (2013). Effects of sports massage and intermittent cold-water immersion on recovery from matches by basketball players. *J. Sports Sci.* 31 11–19. 10.1080/02640414.2012.719241 22935028

[B15] Docherty D. Hodgson M. J. (2007). The application of postactivation potentiation to elite sport. *Int. J. Sports Physiol. Perform.* 2 439–444. 10.1123/ijspp.2.4.439 19171961

[B16] French D. N. Kraemer W. J. Volek J. S. Spiering B. A. Judelson D. A. Hoffman J. R. (2007). Anticipatory responses of catecholamines on muscle force production. *J. Appl. Physiol.* 102 94–102. 10.1152/japplphysiol.00586.2006 16959907

[B17] Gaviglio C. M. Crewther B. T. Kilduff L. P. Stokes K. A. Cook C. J. (2014). Relationship between pregame concentrations of free testosterone and outcome in rugby union. *Int. J. Sports Physiol. Perform.* 9 324–331. 10.1123/ijspp.2013-0106 23881230

[B18] Grasso D. Lanteri P. Di Bernardo C. Mauri C. Porcelli S. Colombini A. (2014). Salivary steroid hormones response to whole-body cryotherapy in elite rugby players. *J. Biol. Regul. Homeost. Agents* 28 291–300. 25001661

[B19] Hodgson M. Docherty D. Robbins D. (2005). Post-activation potentiation: underlying physiology and implications for motor performance. *Sports Med.* 35 585–595. 10.2165/00007256-200535070-00004 16026172

[B20] Kilduff L. P. Finn C. V. Baker J. S. Cook C. J. West D. J. (2013). Preconditioning strategies to enhance physical performance on the day of competition. *Int. J. Sports Physiol. Perform.* 8 677–681. 10.1123/ijspp.8.6.677 23689163

[B21] Kregel K. C. Seals D. R. Callister R. (1992). Sympathetic nervous system activity during skin cooling in humans: relationship to stimulus intensity and pain sensation. *J. Physiol.* 454 359–371. 10.1113/jphysiol.1992.sp019268 1474495 PMC1175609

[B22] Leppäluoto J. Westerlund T. Huttunen P. Oksa J. Smolander J. Dugué B. (2008). Effects of long-term whole-body cold exposures on plasma concentrations of ACTH, beta-endorphin, cortisol, catecholamines and cytokines in healthy females. *Scand. J. Med. Sci. Sports* 68 145–153. 10.1080/00365510701516350 18382932

[B23] Lim I. S. (2016). Correlation between salivary alpha-amylase, anxiety, and game records in the archery competition. *J. Exerc. Nutrition Biochem.* 20 44–47. 10.20463/jenb.2016.0050 28150473 PMC5545204

[B24] Lombardi G. Ziemann E. Banfi G. (2017). Whole-body cryotherapy in athletes: from therapy to stimulation. an updated review of the literature. *Front. Physiol.* 8:258. 10.3389/fphys.2017.00258 28512432 PMC5411446

[B25] McGowan C. J. Pyne D. B. Thompson K. G. Raglin J. S. Osborne M. Rattray B. (2017). Elite sprint swimming performance is enhanced by completion of additional warm-up activities. *J. Sports Sci.* 35 1493–1499. 10.1080/02640414.2016.1223329 27631544

[B26] McGowan C. J. Pyne D. B. Thompson K. G. Rattray B. (2015). Warm-up strategies for sport and exercise: mechanisms and applications. *Sports Med.* 45 1523–1546. 10.1007/s40279-015-0376-x 26400696

[B27] McGowan C. J. Thompson K. G. Pyne D. B. Raglin J. S. Rattray B. (2016). Heated jackets and dryland-based activation exercises used as additional warm-ups during transition enhance sprint swimming performance. *J. Sci. Med. Sport* 19 354–358. 10.1016/j.jsams.2015.04.012 25987491

[B28] Nater U. M. Rohleder N. (2009). Salivary alpha-amylase - a biomarker of the sympathetic nervous system. *J. Psychophysiol.* 46:14. 10.1016/j.psyneuen.2009.01.014 19249160

[B29] Rivera J. Tercero M. J. Salas J. S. Gimeno J. H. Alejo J. S. (2018). The effect of cryotherapy on fibromyalgia: a randomised clinical trial carried out in a cryosauna cabin. *Int. J. Rheumatol.* 38 2243–2250. 10.1007/s00296-018-4176-0 30353267 PMC6223856

[B30] Russell J. M. Birch M. J. Love P. T. Cook P. C. Bracken P. R. Taylor P. T. (2017). The effects of a single whole-body cryotherapy exposure on physiological, performance, and perceptual responses of professional academy soccer players after repeated sprint exercise. *J. Strength Cond. Res.* 31 415–421. 10.1519/JSC.0000000000001505 27227791

[B31] Sands W. A. Kavanaugh A. A. Murray S. R. McNeal J. R. Jemni M. (2017). Modern techniques and technologies applied to training and performance monitoring. *Int. J. Sports Physiol. Perform.* 12(Suppl. 2), S263–S272. 27918664 10.1123/ijspp.2016-0405

[B32] Schaal K. Le Meur Y. Louis J. Filliard J.-R. Hellard P. Casazza G. (2015). Whole-body cryostimulation limits overreaching in elite synchronized swimmers. *Med. Sci. Sports Exerc.* 47 1416–1425. 10.1249/MSS.0000000000000546 25314578

[B33] Serpell B. G. Strahorna J. Colomera C. McKune A. Cook C. Pumpa K. (2018). The effect of speed, power and strength training, and a group motivational presentation on physiological markers of athlete readiness: a case study in professional rugby. *Int. J. Sports Physiol. Perform.* 12 1–15. 10.1123/ijspp.2018-0177 29893598

[B34] Straburzynska-Lupa A. Kasprzak M. P. Romanowski M. W. Kwasniewska A. Romanowski W. Iskra M. (2018). The effect of whole-body cryotherapy at different temperatures on proinflammatory cytokines, oxidative stress parameters, and disease activity in patients with ankylosing spondylitis. *Oxid. Med. Cell Longev.* 2018 1–8. 10.1155/2018/2157496 30402204 PMC6192087

[B35] Strahorn G. J. Serpell L. B. McKune L. A. Pumpa L. K. (2017). Effect of physical and psychosocial interventions on hormone and performance outcomes in professional rugby union players: a systematic review. *J. Strength Cond. Res.* 31 3158–3169. 10.1519/JSC.0000000000002067 28658074

[B36] Szczepanska-Gieracha J. Borsuk P. Pawik M. Rymaszewska J. (2014). Mental state and quality of life after 10 session whole-body cryotherapy. *Psych. Health Med.* 19 40–46. 10.1080/13548506.2013.780130 23535078

[B37] Thomas K. Toward A. West D. J. Howatson G. Goodall S. (2017). Heavy-resistance exercise-induced increases in jump performance are not explained by changes in neuromuscular function. *Scand. J. Med. Sci. Sports* 27 35–44. 10.1111/sms.12626 26639349

[B38] Wozniak A. Mila-Kierzenkowska C. Szpinda M. Chwalbinska-Moneta J. Augustynska B. Jurecka A. (2013). Whole-body cryostimulation and oxidative stress in rowers: the preliminary results. *Arch. Med. Sci.* 9 303–308. 10.5114/aoms.2012.30835 23671442 PMC3648812

[B39] Zalewski P. Klawe J. J. Pawlak J. Tafil-Klawe M. Newton J. (2013). Thermal and hemodynamic response to whole-body cryostimulation in healthy subjects. *J. Cryobio.* 66 295–302. 10.1016/j.cryobiol.2013.03.006 23535554

[B40] Ziemann E. Olek R. A. Kujach S. Grzywacz T. Antosiewicz J. Garsztka T. (2012). Five-day whole-body cryostimulation, blood inflammatory markers, and performance in high-ranking professional tennis players. *J. Athl. Train.* 47 664–672. 10.4085/1062-6050-47.6.13 23182015 PMC3499891

